# eHealth in Self-Managing at a Distance Patients with COPD

**DOI:** 10.3390/life12060773

**Published:** 2022-05-24

**Authors:** Sophie B. Kermelly, Jean Bourbeau

**Affiliations:** 1 Respiratory Division, Department of Medicine, Montreal Chest Institute of the McGill University Health Center, Montreal, QC H4A 3J1, Canada; sophie.bergeronkermelly@mail.mcgill.ca; 2 Respiratory Epidemiology and Clinical Research Unit (RECRU), Center of Outcome and Research Evaluation (CORE), Research Institute of the McGill University Health Centre, 5252 De Maisonneuve, Room 3D.62, Montreal, QC H4A 3S5, Canada

**Keywords:** self-management, behavior change, telemedicine, eHealth, COPD

## Abstract

Worldwide, healthcare delivery for chronic diseases has been challenging due to the current SARS-COV-2 pandemic. The growing use of information and communication technologies via telehealth has gained popularity in all fields of medicine. In chronic respiratory diseases, self-management, defined as a structured but personalized multi-component intervention with the main goal of achieving healthy behavioral change, is an essential element of long-term care. Iterative interventions delivered by a well-trained health coach in order to empower and provide the patient with the tools and skills needed to adopt sustained healthy behaviors have proven to be effective in chronic obstructive pulmonary disease (COPD). Benefits have been shown to both improve patient quality of life and reduce acute exacerbation events and acute healthcare utilization. In COPD, the evidence so far has shown us that remote technologies such as telemonitoring or remote management may improve patient-reported outcomes and healthcare utilization. However, clear limitations are still present and questions remain unanswered. More and better designed studies are therefore necessary to define the place of eHealth in self-managing at a distance in patients with COPD.

## 1. Introduction

Throughout the centuries, there have been sanitary crises, such as the multiple devastating outbreaks of infectious diseases [1]. In 2019, the SARS-COV-2 pandemic turned the world upside down [2,3,4]. As in any crisis, humankind had to adapt to the inevitable changes, creating and innovating to rethink established ways of doing things.

Aside from pandemic-related care, healthcare delivery in chronic diseases has greatly suffered worldwide from the chaos caused by the actual pandemic. Telehealth, defined as the use of information and communication technologies to provide clinical health care through remote methods, has gained popularity in all fields of actual pandemic-era medicine [5]. The use of telemedicine brought its challenges but it has also resulted in several benefits such as a lasting access to healthcare and a reduction in virus transmission among healthcare workers [5].

Self-management interventions, a recognized standard of care in the long-term non-pharmacological treatment of chronic diseases, have now no choice but to innovate in order to remain an integral part of the chronic care follow-up and management [6,7,8]. Management at a distance seems to be a promising way for healthcare professionals to interact and support patients and their families [9]. After more than two years of navigating the changes to health care brought on by the pandemic crisis, we have built and leveraged on our learnings and clinical experience of doing self-management at a distance, supported by growing literature on the subject [10,11,12]. 

This narrative review article mainly focusing on chronic obstructive pulmonary disease (COPD) will (i) define self-management, review its key concepts and showcase its notable success; (ii) give an overview of various methods of self-management at a distance, its implementation and evidence of their benefit; and (iii) address the potential limitations of remote self-management and means of a solution.

## 2. Definition, Key Concepts and Known Successes of Self-Management Intervention 

### 2.1. Definition

**Figure 1:**
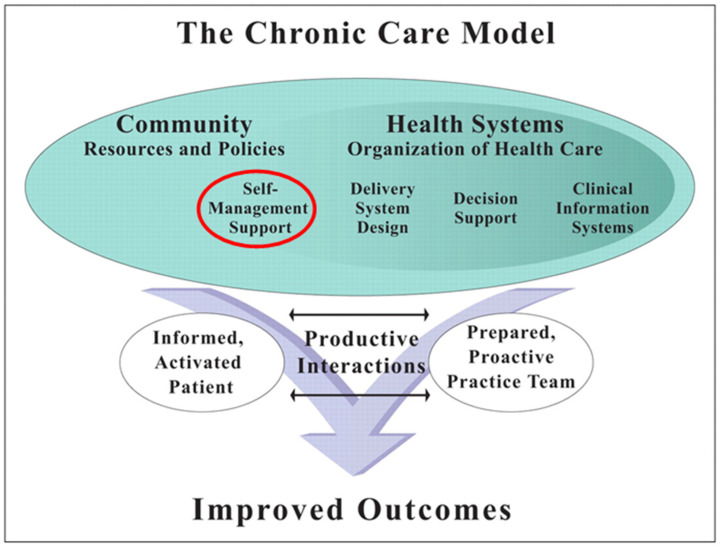
Wagner’s chronic care model. Reprinted/adapted with permission from [13].

Self-management intervention has long since established itself in the management of chronic diseases. The chronic care model, presented in Figure 1, already integrated this concept in 1998, and up until this day, self-management remains an essential component in the comprehensive and optimal management of any chronic disease, including chronic respiratory disease [13]. 

In chronic respiratory diseases, the concept of self-management has developed throughout the years and has been refined over time. In 2016, international experts met and using a Delphi method, they defined a theoretical concept of self-management in COPD and its essential goals and requisites [14]. Self-management has been defined as “an intervention that is structured but personalized and often multi-component” and involves patient-centered interactions. This intervention aims at motivating, engaging and supporting the patients in order for them to gain healthier behaviors and to develop skills to enhance their chronic disease management on a day-to-day basis. 

The ultimate goals of self-management are: (1) optimizing and maintaining physical health; (2) alleviating daily life symptoms and functional incapacities related to their health condition and enhancing emotional and social well-being as well as quality of life; and (3) establishing solid relationships between healthcare professionals, patients and their relatives. 

This conceptual definition leaves room for a personalized implementation of self-management in each care setting and its individualization to every patient. This also allows its application at a distance, as long as the essential components and fundamental objectives remain at the heart of the intervention.

### 2.2. Key Concepts to Success

Self-management interventions in the literature have been very heterogeneous, ranging from supplying an informational pamphlet to developing a complex structure of iterative interventions with well-trained professionals [15,16]. It is therefore important to discuss their basic fundamentals in order to increase the likelihood of providing effective and beneficial self-management strategies.

*Health coach (case manager):* To achieve self-management specific goals, the patient needs to receive continuous and sustained support from a well-trained healthcare professional, acting as a health coach [17]. This health care professional (HCP) has been labeled case manager, navigator or coach. Fundamentally, this role is not simply about transmitting knowledge and educating the patient but understanding the patient’s reality and values in order to empower him. Gaining empowerment is essential in adopting healthier behaviors that will be in line with the patient’s needs and values [18]. Motivational communication, used by the healthcare professional, is a valid technique that every health coach should be trained for [19,20]. This approach is at the center of many self-management programs such as the “Living well with COPD” program (http://livingwellwithcopd.com/, accessed on 18 May 2022) which has demonstrated benefits for the patient and the health care system through randomized controlled trials (RCT) and real-world evidence studies [8,21,22]. 

To fulfill the mandate to accompany the patient toward building self-efficacy and empowerment, a coach (case manager) training must include communication skills such as motivational interviewing [18]. In order to achieve a change in a health behavior, the HCP acting as a case manager needs to fully understand the action planning process. This process, detailed in Table 1, consists of multiple patient-centered steps and strategies to increase the chances of a successful intervention. Experience and training of the case manager may have explained why some studies [23,24] have safely delivered the program and shown a reduction in mortality where in other study there has been report of complications and increased mortality [25]. 

*Behavior change and related self-management strategies:* Promoting healthy lifestyles is an integral part of models of care for individuals with chronic diseases. For patients living with chronic respiratory diseases, smoking cessation (or abstention) and integration of physical activity into daily life are essential components in maintaining control on their chronic illness and improving patient functional capacity and quality of life [26,27,28]. Behavior change can also apply to disease self-management, such as adhering to drug treatment, recognizing and managing an acute exacerbation with a written action plan, or avoiding situations where environmental triggers may be present. Table 2 provides healthy behaviors to achieve in COPD patients and related self-management strategies to adopt. 

**Figure 2:**
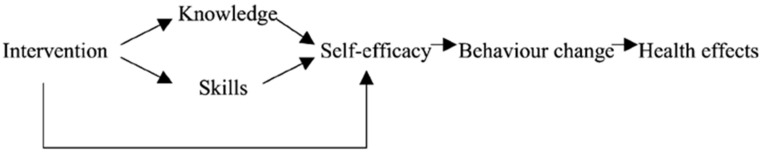
Causal model of behavior change.

The causal model of behavior change summarizes all of the elements necessary for a successful sustained health behavior change process (Figure 2). Patient engagement is the ultimate goal of the HCP interventions in the process of change. 

### 2.3. Evidence of Self-Management Benefit

When the important elements of effective self-management are in place, this method has shown benefits to both the patients receiving it and the health care system. The first landmark multicenter parallel controlled randomized clinical trial showing benefits of self-management in chronic respiratory disease, specifically in COPD, was published in the early 2000s showing significant improvement in health status and reduction in respiratory-related and all-cause hospitalizations and emergency visits [16]. Since that early study, many RCTs evaluating those specific elements of self-management intervention between participants and healthcare provider have been reviewed and published in a most recent Cochrane systematic review with meta-analysis, again supporting these positive results [8]. Self-management interventions reduced respiratory-related hospitalizations and improved health related quality of life. No excess mortality risks were observed, which strengthens the view that COPD self-management interventions are unlikely to cause serious adverse events. However, long-term sustainability is unknown since trials have usually not followed-up patients over 12 months.

To put emphasis on which component of self-management interventions are responsible for the effectiveness, a meta-analysis on the subject helped us to better understand this complex intervention [16]. Overall, we learned it requires at least an iterative process of interactions between participant and HCP, and ideally also included formulation of goals and provision of feedback. A written action plan in the event of an exacerbation and teaching on how to use it appropriately has been proven effective. Content could be delivered verbally as written material (hardcopy or digital) or via audiovisual media. 

Knowing the beneficial effects of self-management, its delivery, either remotely or in person, now needs to aim high and reach a great number of patients suffering from chronic respiratory diseases that can clearly benefit from this strategy. The question remains as to the utility and the evidence that e-health in self-management interventions add to the known benefit with respect to the process, the patient behavior and patient outcomes. 

## 3. Self-Management over Time and at a Distance

In the early days of self-management in chronic respiratory diseases, that is, at the beginning of the 2000s, trials used minimal technologies to teach and support self-management interventions at a distance. Telephone follow ups were the most common use of technology, in addition to standard in-person visits [8,16].

In the last decade, there has been an increased interest in health information technology (eHealth) to ease the working life of health practitioners and having the potential to transform the way patients are monitored and healthcare is delivered. There have been big promises made for the use of eHealth, with the rapid development of information and communication technologies for the management of chronic diseases. Specifically, in the field of respirology, COPD has been the most studied chronic respiratory disease at a distance [11,12,30,31]. Considering the tracking of COPD patients for earlier detection of exacerbations as well as early interventions to minimize the deterioration that so frequently results in the need for hospitalizations, have the promises been delivered? 

### 3.1. Use of Health Information Technology and at a Distance Support of COPD Patients 

*Remote monitoring interventions without component of self-management intervention:* Telehealth encompasses a wide variety of technologies and interventions and many applications used for the management of COPD [31]. Table 3 presents studies that have used various health information technology methods at a distance and its potential utility in COPD. Some can include remote monitoring technology, which requires daily use of a laptop or a tablet with monitoring equipment, with results received by the healthcare professional. Typical monitoring equipment could include a sphygmomanometer (to measure blood pressure and heart rate), a pulse oximeter (to measure oxygen levels in the blood), a spirometer (to measure lung function), a thermometer, and other devices. Interventions were not specifically self-management interventions but could involve regular phone calls with healthcare professionals for patients to talk about their symptoms and completion of health questionnaires. 

Remote monitoring or remote consultation plus usual care or remote monitoring alone was no better than usual care [31]. Multi-component interventions with asynchronous remote monitoring were no better than usual care although it results in some cases in fewer re-admissions to hospital. However, it is unclear if the reduction in hospitalization was due to the remote monitoring. Furthermore, the long-term effect was not known. We can conclude that remote interventions for monitoring or remote consultations without component of self-management interventions have not demonstrated to be clinically effective in people with COPD. 

*“Stand alone” physiology telemonitoring:* “Stand alone” physiology telemonitoring without daily monitoring of symptoms has not demonstrated benefit. Two studies, PROMETE II and CHROMED, have been conducted using physiology telemonitoring for overseeing patients and for enabling HCPs to promptly intervene in the setting of an acute exacerbation [32,33]. In PROMETE II, a multicenter randomized 12-month trial, the system included a remote patient-monitoring platform with equipment provided for blood pressure, oxygen saturation, heart rate and spirometry measurement, which were actively measured by the patient at home as per instructions. Respiratory rate and oxygen adherence data were passively collected by a device connected to the oxygen feed from their main oxygen source [32]. This trial comparing the monitoring system to routine clinical practice in patients with severe COPD showed no reduction of COPD-related emergency department (ED) visits or hospital admissions. In CHROMED, the novel feature of monitoring forced oscillation technique (FOT) was compared with usual care in a randomized clinical trial [33]. Using “stand alone” physiology telemonitoring with FOT did not result in a reduction on time to first hospitalization or improvement in health-related quality of life. 

*Home telemonitoring trials in COPD that included monitoring of patient symptoms:* A systematic review of home telemonitoring trials in COPD, showed a positive effect in reducing exacerbations and hospital admissions [34]. In this review, the main limitation was that none of the studies were designed to demonstrate additional benefit of monitoring respiratory physiology to monitoring symptoms. The benefits seen in these studies could well be related only to daily monitoring of symptoms with self-management and that physiology monitoring has no added value. Furthermore, although patients were generally satisfied and found the tele-monitoring systems useful to help them manage their disease, they reported some difficulties in their use, sometimes related to lower compliance rates [35]. It is unknown to how many patients and which patients the physiology monitoring was not acceptable. 

### 3.2. Information and Communication Technologies for Self-Management Interventions and Impact on Health and Hospital Service Uses

Digital technology through a broad variety of methods to virtually deliver self-management interventions has a potential to assist patients to manage their condition, including essential skills such as problem solving; decision making; resource utilization; to form a partnership between patient and HCP; and to act to prevent a deterioration or to self-care for a condition that is worsening. 

In 2015, a joint American and Canadian thoracic society guideline was published aiming at the prevention of acute exacerbations among COPD patients. This guideline assesses pharmacological and non-pharmacological therapy such as tele-health with self-management interventions [36]. The studies of “tele-health with self-management interventions” to be included in the meta-analysis had to be a randomized clinical trial and the intervention defined as remote that comprises the following elements: (a) electronic transfer of self- report data over a distance; (b) use of a device located in the patient’s home or on her/his person (mobile device); and (c) personalized feedback from a healthcare professional who exercises their skills and judgement in the provision of tailored advice to the patient or automated feedback based on a pre-determined algorithm. The recommendation was based on three RCTs from one systematic review that met the definition and 18 additional RCTs. Of these, only six studies of 707 subjects were poolable for meta-analysis. They examined the outcomes on the number of ED visits, exacerbations, and hospitalizations. No statistically significant results were found for any of these outcomes. Furthermore, the variability among the telemonitoring applications precludes accurate comparison between studies. Reviews were not reporting negative effects, suggesting that telehealth is a safe option for delivery of self-management support. The conclusion that we can make with caution is that there is insufficient evidence to support the contention that virtually deliver and support self-management prevents COPD exacerbations. 

Since then, one meta-analysis compared self-management interventions delivered by computer or mobile technology versus face-to-face interventions among moderate to very severe COPD [30]. This review only included three randomized controlled trials but 1580 participants. The effect of smart technology on self-management, symptoms and health status up to 6 months into the trials was significantly better than when participants received face-to-face/digital and/or written support for self-management of COPD (SMD −0.22, 95% confidence interval (CI) −0.40 to −0.03; *p* = 0.02). However, at 12 months there was no significant between-group differences although this longer term follow up was reported in only one trial. The three studies in this meta-analysis were at high risk of bias, of poor quality and insufficient for advising about the health benefits of using smart technology as an effective means of supporting, encouraging, and sustaining self-management. 

### 3.3. Telehealth-Supported Components of Self-Management and Their Impact on Patient Behaviors and the Process of Self-Management

There has been a lack of a suitable tool to analyze the important components of self-management interventions, which has justified the development of the PRISMS taxonomy of self-management support [37]. Many components could be potentially delivered via telehealth, and have been grouped under the following:

1. Patient education and information provision;
2. Remote monitoring with feedback and action plans (e.g., peak expiratory flow or blood glucose monitoring with action plans);
3. Telehealth-facilitated clinical review;
4. Adherence support (e.g., medication or lifestyle intervention adherence);
5. Psychological support;
6. Lifestyle interventions (e.g., smoking cessation, exercise, weight loss.

A metareview has been published with synthesized systematic review evidence on telehealth interventions to support self-management in a variety of chronic diseases including COPD [38]. Included systematic reviews of the interventions had multiple components but no single self-management component was found to be consistently effective, or consistently ineffective. We are reporting on the evidence specific to some components of self-management interventions delivered by tele health.

Education and information: The evidence neither supports nor refutes the linking of telehealth interventions with increased knowledge or self-care behaviors, but the studies were of poor methodological quality.

Monitoring and feedback: This was associated with improved clinical outcomes in diabetes and heart failure but findings for asthma and COPD were mostly neutral or inconsistent. 

Facilitation of remote clinical review: Teleconsultations, videoconferencing, and telephone follow-up designed to review symptoms or clinical course are important aspects of telehealth interventions. However, from this review, in asthma and COPD, the findings were typically neutral.

Adherence support and lifestyle interventions: Achieving improved clinical outcomes with such interventions may be challenging, as they involve significant behavior change. There are studies reporting that interventions improve medication adherence. 

Multicomponent and intensive interventions: Most telehealth interventions are complex multicomponent interventions, but most studies either provided limited description of the interventions or did not specifically analyze the impact of individual components on the efficacy of the intervention as a whole. Same for the intensity of the interventions which varies widely, and few reviews specifically analyzed the relationship between the intensity (in terms of either contact with health care professionals or the complexity or number of components in the intervention) and outcomes. 

Another meta-analysis aimed at assessing the potential benefit and harm of digital technology on health behaviors and encouraging patient engagement. Fourteen studies were included in the meta-analyses (1518 participants) ranging from a 13-week to a 52-week duration. Participants had mild to very severe COPD. The systematic review included studies with digital technology (mobile phone for three studies, smartphone applications for one study, and web or internet-based for five studies) with or without routine supported self-management or multi-component interventions with short messaging services for reminders, education, motivation or prevention (digital components such as mobile phone for one study, and web or internet-based for four studies). No statistically significant difference was demonstrated for exercise capacity, self-efficacy, exacerbations and adverse effects. Only the multi-component intervention studies showed statistically significant improvement in HRQoL [12]. Unfortunately, although the intent was to assess health behavior as the primary outcome, due to a lack of data, this systematic review did not really assess health behavior. Risk of bias was high and GRADE levels of evidence were low to very low due to lack of blinding and imprecision. Currently there is no guidance for interpreting behavior change technique components of a digital intervention for changes in health outcomes. 

## 4. Potential Limitations to Self-Management at a Distance

Despite many advances in the recent decades regarding self-management, delivering self-management at distance using digital technology raises many unanswered questions and brings about important limitations that mandate a reflection.

Those limitations include: (i) what mHealth adds to the already recognized self-management education intervention with respect to patient intrinsic motivation, self-efficacy and behavior changes; (ii) what are effective structures or strategies for delivery of such interventions owing to the scarcity of high-quality studies and suboptimal reporting quality of the reviewed papers; (iii) how can we generalize to the various severity of a chronic disease (mild, moderate, severe) or to patients with comorbidities.

The use of technology should help with empowering patients to integrate new practices and routines to deal with the daily rigors of COPD self-management, such as monitoring symptoms, physical activity and medication taking. Technological interventions should facilitate the process of self-care by supporting patients, both educationally and motivationally, in their day-to-day decision making. Incorporation of timely clinical information not only increases the patient’s ability to recognize opportunities for intervention, but also increases the patient’s quick reaction, and allows for immediate meaningful feedback. Future research should provide clear information on how self-management is assessed and should include measures that allow comments on behavioral change.

Furthermore, if we are to telemonitor respiratory physiology, parameters detecting exacerbations and recovery should drive the agenda of researchers and those in the field of technology discovery. A better understanding of physiological changes can be studied with the use of wearable technology. As we are waiting for new knowledge, we should build on what we already know in successful COPD self-management interventions. We may be better right now using eHealth to increase patient activation and empowerment and to facilitate collaboration between patients and HCPs. 

There is also need for additional research to determine the optimal structure, format, and delivery methods for educational instructions that are used in mHealth interventions for patient education and aiming at effective self-management. There is a need to adopt standard tools, for reporting mHealth quality evaluation. However, overall there is a need to know exactly what telehealth interventions bring as positive contribution to already recognized benefits of self-management interventions. As an objective to standardize and to structure research studies around mHealth, the World Health Organization (WHO) has developed criteria checklist guidelines: the mERA essential criteria [39]. The adoption of those standardized tools could help future research to overcome its shortcomings. 

## 5. Conclusions

The impact of telehealth-supported self-management on disease control and health care utilization often has shown no effect and/or inconsistent effects. We have enough evidence guiding us in “doing it right”, i.e., doing self-management interventions right. However, we still do not know “doing the right thing”: the role of eHealth in self-managing at a distance patients with COPD. 

Now more than ever, with the multiple changes associated with the COVID-19 pandemic era, the development and use of technologies to enable and promote remote monitoring, provide information and communicate with the patients for more effective self-management is of primary importance. Although more research in that field is needed, a return to the days without telecommunication in health is highly unlikely. Thus, we need to continue to adapt our way of doing things for this new and improving standard of care. Pre-defined tags in self-management at a distance are therefore necessary to continue to evolve and innovate. If we really want to make progress, eHealth should be embedded into regular care as an adjunct or enhancement to current self-management interventions with better study quality. Furthermore, since COPD is a chronic disease, long-term involvement seems crucial.

Finally, we do not need more studies, but rather better and larger-scale trials of self-management interventions delivered by tele-health, based on explicit self-management theory, linked with an evaluation that include detailed description of the intervention and the process delivery, intermediate outcomes such as self-efficacy and specific behavior changes, in order to gain more insight into the optimal combination of in person care and eHealth-based self-management. Additionally, there is a need for studies to assess the preferences of various patients, the best platform for patients that is easy to use and the related costs.

## Figures and Tables

**Table 1:** Action planning process to achieve a successful self-management intervention.

Possible Gaps in the Delivery Style	Action Planning Points for Success
The patient comes to the education session, and he is presented with a predetermined, inflexible agenda	Action planning is patient centered Focus on what the patient wants and not what the patient is told to do!
The patient receives a plan for change, because “it is important,” although he has not had a chance to participate in its development	2. Action planning is collaborative Behavior change is more likely to take place and be sustained when the patient is engaged and participates in problem-solving and in the decision-making process
The patient can see the final objective; however, no concrete, realistic plan to achieve it has been set up	3. Action planning is SMART: specific, measurable, achievable, relevant, and timed
It is unknown whether the patient is motivated or not, and whether he feels confident to integrate the new behaviors, or he perceives there are barriers	4. Action planning includes the evaluation of self-efficacy
The patient leaves the session with a behavior change plan, but he does not know in which way his progresses will be evaluated and adjusted	5. Action planning includes arranging for follow-up
The patient leaves the session with a behavior change plan, but he is left to implement it by himself, without receiving feedback or recognition from others	6. Action planning includes a final commitment statement: the “contract”, signed between the patient and his case manager or a family member. This will help predict the success, as there is accountability

Reprinted/adapted with permission from [18].

**Table 2:** Self-management skills and strategies for healthy behaviors in COPD patients.

Healthy Behavior	Self-Management Skill (Strategy)
Live in a smoke-free environment Comply with your medication	Quit smoking, remain nonsmoker, and avoid second-hand smoke Take medication as prescribed on a regular basis and use proper inhalation techniques
Manage to maintain comfortable breathing	Use according to directives: The pursed-lip breathing technique The forward body position
Conserve your energy Manage your stress and anxiety	Prioritize your activities, plan your schedule, and pace yourself Use your relaxation and breathing techniques, try to solve one problem at a time, talk about your problems and do not hesitate to ask for help, and maintain a positive attitude
Prevent and seek early treatment of COPD exacerbations	Get your flu shot every year and your vaccine for pneumonia Identify and avoid factors that can make your symptoms worse Use your plan of action according to the directives (recognition of symptom deterioration and actions to perform) Contact your resource person when needed
Maintain an active lifestyle	Maintain physical activities (e.g., activities of daily living, walking, climbing stairs) Exercise regularly (according to a prescribed home exercise program)
Keep a healthy diet	Maintain a healthy weight, eat food high in protein and eat smaller meals more often (5–6 meals per day)
Have good sleep habits	Maintain a routine, avoid heavy meals or stimulants, and relax before bedtime
Maintain a satisfying sex life	Use positions that require less energy Share your feelings with your partner Do not limit yourself with intercourse, create a romantic atmosphere Use your breathing, relaxation, coughing techniques
Get involved in leisure activities	Choose leisure activities that you enjoy Choose environments in which your symptoms will not be aggravated Pace yourself through the activities while using your breathing techniques Respect your strengths and limitations

Reprinted/adapted with permission from [29].

**Table 3:** Studies that have used various methods of remote self-management and its potential utility in COPD.

Methods	Studies (n)	Examples of Its Potential Utility
Telemonitoring via wireless or corded medical device	4	Smart mobile health tool for self-management of COPD exacerbations: Mobile phone spirometer Pulse oximeter Forehead thermometer
Smartphone applications	1	Smartphone application-based self-management program: Smartphone app (self-monitoring, recording exercise data, symptoms, bronchodilator use, healthcare use due to exacerbations) Educational materials Pedometer Weekly group education and exercise sessions in the first month Prescribed individualized exercise sessions Communication via phone or messaging research team and other
Web/Internet-based platforms	9	Home-based PLB re-enforcement sessions via video conference Single exposure to educational materials viewed at the clinic Clinical video physician-led video, providing clinical information about COPD symptoms and self-management strategies Lay video included patients’ role playing a scenario offering opinions and narratives about COPD self-management multi-component home-based COPD disease management: Components included self-management program, home monitoring, and e-health telephone/web platform Self-management program was based on “Living Well with COPD” program Participants completed telephone questionnaire Participants recorded days they experienced worsening symptoms E-health telephone/web platform allowed timely participant follow-up for early detection of potential exacerbations and worsening symptoms

Reprinted/adapted with permission from [12].

## Data Availability

Not applicable.
